# Impaired bile acid metabolism with defectives of mitochondrial-tRNA taurine modification and bile acid taurine conjugation in the taurine depleted cats

**DOI:** 10.1038/s41598-020-61821-6

**Published:** 2020-03-18

**Authors:** Teruo Miyazaki, Sei-Ich Sasaki, Atsushi Toyoda, Fan-Yan Wei, Mutsumi Shirai, Yukio Morishita, Tadashi Ikegami, Kazuhito Tomizawa, Akira Honda

**Affiliations:** 1 Joint Research Center, Tokyo Medical University Ibaraki Medical Center, Ibaraki, 300-0395 Japan; 2 Ibaraki Prefectural University of Health Sciences, Ibaraki, 300-0394 Japan; 3 Toyo Public Health College, Tokyo, 151-0071 Japan; 4 College of Agriculture, Ibaraki University, Ibaraki, 300-0393 Japan; 5 Department of Molecular Physiology, Faculty of Life Sciences, Kumamoto University, Kumamoto, 860-8556 Japan; 6 Diagnostic Pathology Division, Tokyo Medical University Ibaraki Medical Center, Ibaraki, 300-0395 Japan; 7 Department of Internal Medicine, Division of Gastroenterology and Hepatology, Tokyo Medical University Ibaraki Medical Center, Ibaraki, 300-0395 Japan

**Keywords:** Metabolomics, Nutrition disorders, Hepatology

## Abstract

Taurine that conjugates with bile acid (BA) and mitochondrial-tRNA (mt-tRNA) is a conditional essential amino acid in humans, similarly to cats. To better understand the influence of acquired depletion of taurine on BA metabolism, the profiling of BAs and its intermediates, BA metabolism-enzyme expression, and taurine modified mt-tRNAs were evaluated in the taurine deficient diet-supplemented cats. In the taurine depleted cats, taurine-conjugated bile acids in bile and taurine-modified mt-tRNA in liver were significantly decreased, whereas unconjugated BA in serum was markedly increased. Impaired bile acid metabolism in the liver was induced accompanied with the decreases of mitochondrial cholesterol 27-hydroxylase expression and mitochondrial activity. Consequently, total bile acid concentration in bile was significantly decreased by the low activity of mitochondrial bile acid synthesis. These results implied that the insufficient dietary taurine intake causes impaired bile acid metabolism, and in turn, a risk for the various diseases similar to the mitochondrial diseases would be enhanced.

## Introduction

Taurine (β-aminoethanesulphonic acid) is an amino acid derivative contained abundantly in most tissues and cells^1–4^. It is considered an essential amino acid for the development and growing periods of most animals because of an immature ability to biosynthesize it from cysteine in the liver. In humans, the ability to biosynthesize taurine is limited because of the very low activity and adaptation of the two key enzymes of taurine biosynthesis^5^; cysteine dioxygenase and cysteine sulfinate decarboxylase^6,7^. In adults, taurine is maintained by sufficient ingestion of animal protein food and by biosynthesis capacity; however, taurine depletion, which usually manifests in low levels in blood and urine, occurs with long-term parental nutrition^8^, strict vegan diets^9^, and a variety of clinical conditions such as short bowel syndrome^10^, intensive chemotherapy^11^, whole-body irradiation^11,12^, hepatic diseases^13,14^, and chronic renal failure^15,16^. Therefore, taurine is a conditionally essential amino acid for humans. The importance of taurine homeostasis in the body has been supported in the taurine depletion experimental models. Critical developmental failures and dysfunction in various tissues have been observed in taurine transporter knockout mice^17–20^. However, the taurine depletion in the knockout mice was congenitally caused, and the taurine depletion model in rodents is hardly developed by a dietary deficiency of taurine because of the higher ability of taurine biosynthesis^4^.

Cats have a low taurine biosynthesis ability by the limited-expression and activity of the key enzymes^5^ similar to humans and have a low ability of renal regulation against fluctuation of taurine in the body^7^. Therefore, cats have been used for an established experimental model^21^ as the dietary induced-acquired taurine depletion with developments of certain diseases including blindness^22^ and expanded cardiomyopathy^23^. Taurine has numerous physiological and pharmacological roles in various tissues^21,24–27^. One of the most established biological actions of taurine is its conjugation with bile acids (BAs) to promote bile excretion via enhancing their water solubility into bile, and consequently, efficiently facilitating the absorption of lipids and lipid-soluble vitamins^28,29^. In the cats, BAs in the bile are exclusively conjugated with taurine^30^; the percent distribution ratio of the major BA types is 98.4% of taurine-conjugated form (79.1% of cholic acid; CA, 19.3% of deoxycholic acid; DCA), 1.1% of glycine-conjugated CA, and 0.5% of unconjugated CA in the cats fed with commercial diet^31^. In the chronic taurine-deficient diet fed cats, the unconjugated BAs in the bile was increased with decreased taurine conjugated BAs, but conversion to glycine conjugated form did not occur^7,31^, and furthermore, the biliary total bile acid (TBA) concentration was significantly decreased to 44%^7^. Thus, both the concentrations of taurine conjugated BAs and total BA were markedly decreased in the taurine depleted cats^7,31^.

In addition, taurine has been identified in modified mitochondrial (mt)-tRNAs containing 5-taurinemethyluridine (*τ*m^5^U) and 5-taurinemethyl 2-thiouridine (*τ*m^5^s^2^U)^32^. Impaired taurine modification with mt-tRNAs causes certain inherited mitochondrial diseases; mitochondrial encephalopathy, lactic acidosis, and stroke-like episodes (MELAS); and myoclonus epilepsy with ragged red fibers (MERRF)^33^. Mitochondrial diseases occur because of specific point mutations in the region of mitochondrial deoxyribonucleic acid (mt-DNA) that codes for tRNAs, although impaired taurine modification with mt-tRNAs may also be caused by taurine depletion^33,34^. Therefore, taurine depletion may impact BA conjugation, as well as BA synthesis, because mitochondrial metabolism is involved in BA synthesis.

We hypothesized that insufficient nutritional taurine ingestion may influence the metabolism of BAs that regulates the absorption and metabolism of lipids and lipid-solved vitamins, and the consequent abnormalities in BA metabolism may be associated with various symptoms caused by taurine deficiency. In the present study, we examined the influence of acquired taurine depletion induced by insufficient ingestion on BA metabolism in the liver in cats, which have no ability to biosynthesize taurine.

## Results

### Taurine concentrations in serum and liver, body and tissue weights, and serum biochemical data

**Figure 1:**
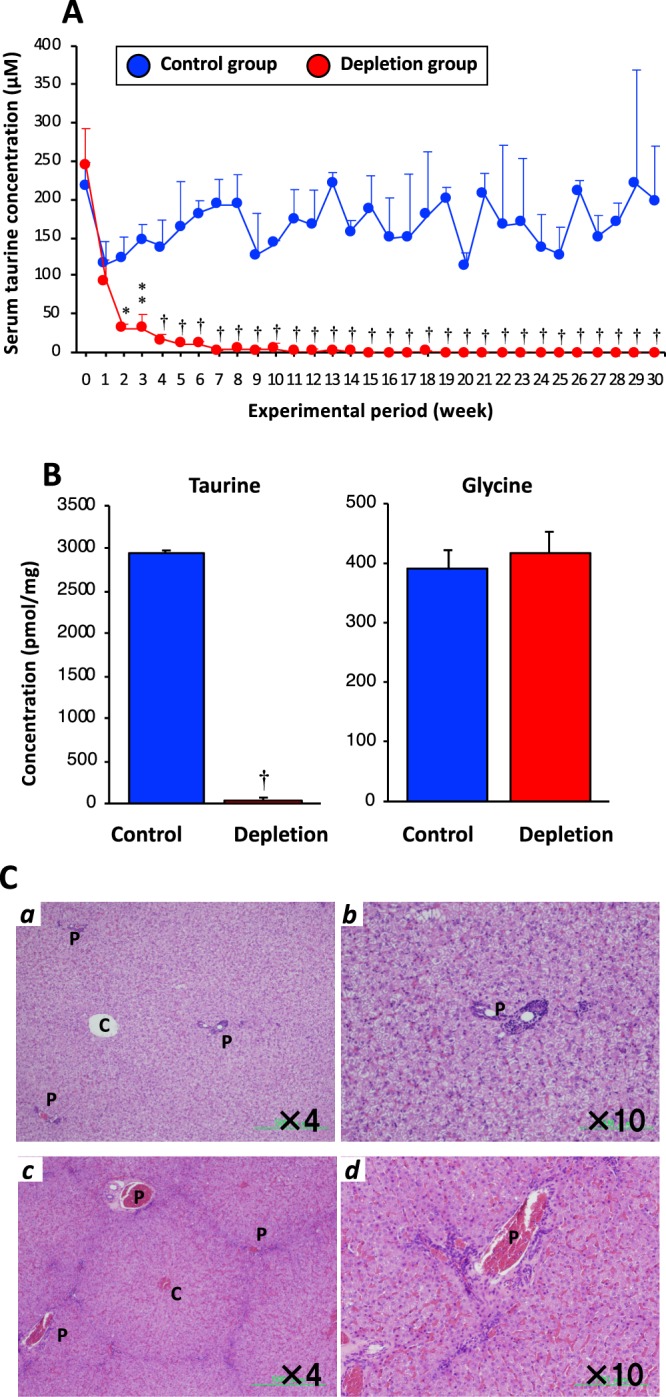
Taurine concentration in serum and liver, and histological observations of hepatic tissue. (**A**) Progress of serum taurine concentration during the experimental diet feeding for 30 weeks. (**B**) Taurine and glycine concentrations in the liver after the 30-week feeding. (**C**) H&E stain of hepatic tissue; (**C**a & **C**b) Overall hepatic lobule (objective × 4) and the portal region (×10) images in the Control group. (**C**c & **C**d) Overall hepatic lobule (×4) and the portal region (×10) images in the Depletion group. *C* and *P* in Fig. 1**C**b and **C**d show the central vain and portal vain, respectively. Data are the mean ± SE. *P < 0.05, **P < 0.01, ^†^*P* < 0.001 versus the Control group at each point by unpaired Student *t*-test.

During the 30-week experimental diet feeding period, the serum taurine concentration in the taurine-depletion (Depletion) group fed the taurine-deficient diet had decreased to one half of the level after 1 week and significantly decreased after 2 weeks, compared to the concentrations in the control (Control) group fed the taurine-supplemented diet (Fig. 1A). After 7 weeks, the serum taurine in the Depletion group was undetectable (Fig. 1A). Taurine concentration in the liver decreased to mostly undetectable in the Depletion group; however, glycine concentration between the two groups were not significantly different (Fig. 1B). The two groups did not differ in body weight (BW) change during the feeding period and did not differ in the tissue weight/BW ratio of the liver, spleen, and heart after the experimental period (Supplemental Fig. 1C,D).

After 30 weeks, the serum levels of glucose, total-cholesterol, and high-density lipoprotein (HDL)-cholesterol were significantly lower in the Depletion group than in the Control group (Table 1). The levels of alanine aminotransferase (ALT), aspartate aminotransferase (AST), free fatty acid (FFA), triglyceride, alkaline phosphatase (ALP) phospholipids, and total protein were not significantly different between the two groups.

**Table 1:** Body and liver tissue weights in the taurine-depletion cats after feeding of the experimental diets for 30 weeks.

	Control group (*n* = 4)	Depletion group (*n* = 6)
***Weight***		
Body (kg)	3.1 ± 0.5	3.2 ± 0.4^*ns*^
Liver (g)	80.6 ± 13.5	75.7 ± 10.8^*ns*^
***Blood biochemical paramete****r*		
ALT (IU/L)	56.3 ± 14.7	46.7 ± 9.6^*ns*^
AST (IU/L)	83.8 ± 9.1	62.7 ± 16.9^*ns*^
Glucose (mg/dL)	484.4 ± 55.8	271.6 ± 55.7*
FFA (µEq/L)	385.7 ± 40.5	463.0 ± 225.1^*ns*^
Total-Chol (mg/dL)	66.2 ± 7.9	35.3 ± 6.2*
HDL-Chol (mg/dL)	39.7 ± 19.3	10.3 ± 9.5*
TG (mg/dL)	95.4 ± 42.0	69.8 ± 29.0^*ns*^
ALP (IU/mL)	12.0 ± 2.9	15.2 ± 4.1^*ns*^
C4 (ng/mg)^**§**^	5.4 ± 1.6	22.9 ± 6.5*
4βHC (ng/mg)^**§**^	28.8 ± 2.1	27.9 ± 1.1^*ns*^
25HC (ng/mg)^**§**^	6.1 ± 2.9	5.0 ± 2.8^*ns*^
Sitosterol (µg/mg)^**§**^	7.8 ± 0.7	4.6 ± 0.9*
Phospholipid (mg/dL)	50.6 ± 30.7	49.6 ± 18.2^*ns*^
Total-protein (g/dL)	14.6 ± 1.1	14.6 ± 1.1^*ns*^

Note: Data are shown as the mean ± SE. *P < 0.05 versus the Control group by unpaired Student’s *t*-test. ^§^Oxysterols are expressed per Total-Chol (mg). *Abbreviations*: *4βHC*; 4β-hydroxycholesterol, *25HC*; 25-hydroxycholesterol, *ALP*; alkaline phosphatase, *ALT*; alanine aminotransferase, *AST*; aspartate aminotransferase, *C4*; 7α-hydroxy-4-cholesten-3-one, *FFA*; free fatty acids, *ns*; no significant difference, *TG*; triglyceride, *Total-Chol*; total cholesterol.

### Histological evaluation of hepatic tissue

In the hematoxylin and eosin (H&E) stain images of the liver, infiltration of inflammatory cells such as lymphocyte, eosinophil, and neutrophil aggregates existed around the periportal regions and in areas between the portal veins in the Depletion group (Fig. 1Cc,Cd), whereas these abnormalities did not exist in the Control group (Fig. 1Ca,Cb).

In the histological and macroscopic examinations of the heart and eyes, several abnormalities such as pupil dilation and a cloudy eyeball (Supplemental Fig. 1A) and expansion of the atria and ventricles in the heart (Supplemental Fig. 1B) were similar to findings in previous studies reporting retinal degeneration^22^ and cardiomyopathy^23^ in taurine-depleted cats.

### Bile acid profiles in bile

**Figure 2:**
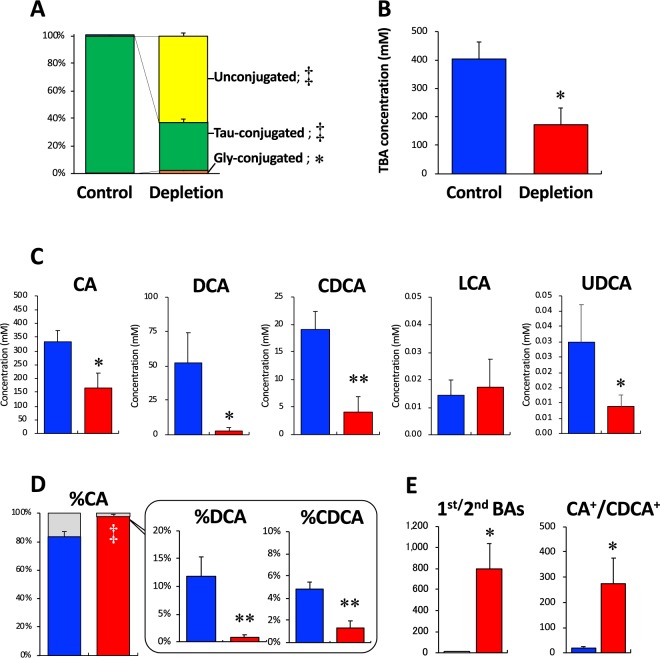
Bile acid profiles in bile. (**A**) Proportion of unconjugated and taurine/glycine-conjugated forms of BAs (the sum of all BA types). (**B**) TBA concentration. (**C**) Concentrations of the respective types of BAs (the sum of unconjugated and conjugated forms). (**D**) Proportion of BA types (the sum of unconjugated and conjugated forms). (**E**) Ratios of the primary BAs to the secondary BAs (1^st^/2^nd^ BA) and the CA-derived BAs to the CDCA-derived BAs (CA^+^/CDCA^+^) are presented. Data are the mean ± SE.*P < 0.05, **P < 0.01, ^‡^*P* < 0.0001 versus the Control group by unpaired Student *t*-test. *Abbreviations*: *BA;* bile acid, *CA*, cholic acid; *CDCA*, chenodeoxycholic acid, *Control*; the taurine-contained diet supplemented group; *DCA*, deoxycholic acid, *Depletion*; the taurine-deficient diet supplemented group, *Gly-conjugated*; the sum of glycine conjugated bile acids, *LCA*, lithocholic acid; *Tau-conjugated*; the sum of taurine conjugated bile acids, *TBA;* total bile acid, *UDCA*, ursodeoxycholic acid, *Unconjugated*; the sum of unconjugated bile acids.

After the feeding period, more than 99.9% of BAs in the bile of the Control group was conjugated with taurine, whereas the glycine-conjugated and unconjugated forms of BA were less than 0.1% (Fig. 2A). In the Depletion group, taurine-conjugated BA was reduced to less than 40%, and the unconjugated form was increased to more than 60% (Fig. 2A). Glycine-conjugated BA was increased to only >2% (Fig. 2A). These differences in the all three BA forms between the two groups were significant. The TBA concentration of bile in the Depletion group was significantly decreased to less than one-half that in the Control group (Fig. 2B). Biliary levels of all types of BA, except for lithocholic acid (LCA) in the Depletion group, were significantly decreased, compared to the levels in the Control group (Fig. 2C). In the Control group, the proportion of CA, DCA, and chenodeoxycholic acid (CDCA), which were the sum of the unconjugated and conjugated forms, was approximately 80%, 10%, and 5%, respectively (Fig. 2D). In the Depletion group, CA was significantly increased to >98%, whereas CDCA and DCA were significantly decreased, compared to the levels in the Control group (Fig. 2D). The levels of LCA and ursodeoxycholic acid (UDCA) in both groups were less than 0.01%. The ratio of CA-derived BAs (CA and its secondary bile acid DCA) to CDCA-derived BAs (CDCA and its secondary bile acids LCA and UDCA) and the ratio of the primary BAs (CA + CDCA) to the secondary BAs (DCA + LCA + UDCA) were significantly higher in the Depletion group than in the Control group (Fig. 2E).

### Bile acid profiles in peripheral blood

**Figure 3:**
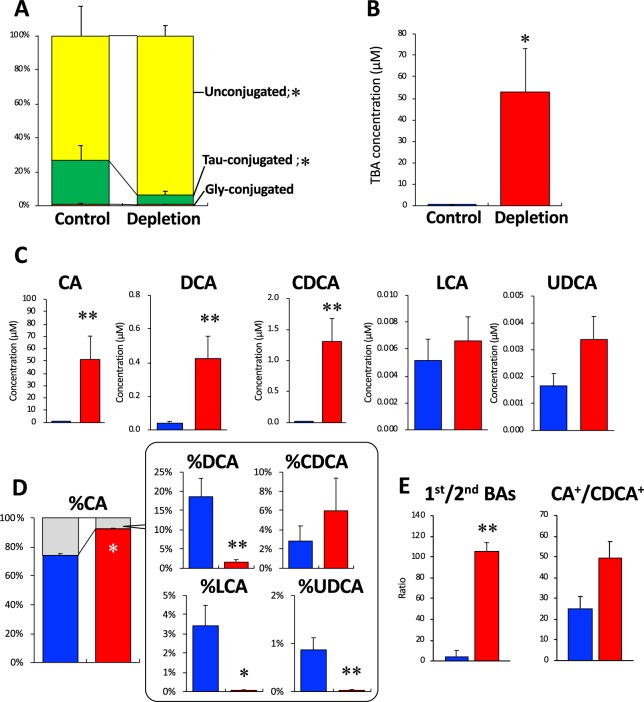
Bile acid profiles in serum. (**A**) Proportion of unconjugated and taurine/glycine-conjugated forms of BAs. (**B**) TBA concentration. (**C**) Concentrations of the respective types of BAs. (**D**) Proportion of BA types. (**E**) Ratios of 1^st^/2^nd^ BA and CA^+^/CDCA^+^. Data are the mean ± SE. *P < 0.05, **P < 0.01 versus the Control group by unpaired Student *t*-test. *Abbreviations*: See the legend of Fig. 2 for abbreviations.

In serum, the BA proportions of unconjugated and taurine-conjugated forms were approximately 75% and 25%, respectively, in the Control group; however, these forms were significantly increased to 95% and significantly decreased to 5.5%, respectively, in the Depletion group (Fig. 3A). The glycine-conjugated forms in the both groups were less than 1% and were not significantly different. The serum TBA concentration was >200-fold higher in the Depletion group than in the Control group (53.0 ± 20.2 vs. 0.2 ± 0.08 µM, *P* < 0.05; Fig. 3B). Serum concentrations of CA, DCA, and CDCA were significantly increased in the Depletion group, compared to the concentrations in the Control group (Fig. 3C). The proportions of CA, CDCA, DCA, LCA, and UDCA in the serum were approximately 75%, 3%, 20%, 3%, and 1%, respectively, in the Control group (Fig. 3D). In the Depletion group, the primary bile acids CA and CDCA were increased to approximately 90% and 6%, respectively. However, the percentage of the secondary BAs were significantly decreased in the Depletion group, compared to the Control group (Fig. 3D). The primary/secondary BA ratio was significantly higher in the Depletion group than in the Control group, although the CA-derived BAs/CDCA-derived BAs ratio was not significantly different between the two groups (Fig. 3E).

### Cholesterol and oxysterol concentrations in the liver and serum

**Figure 4:**
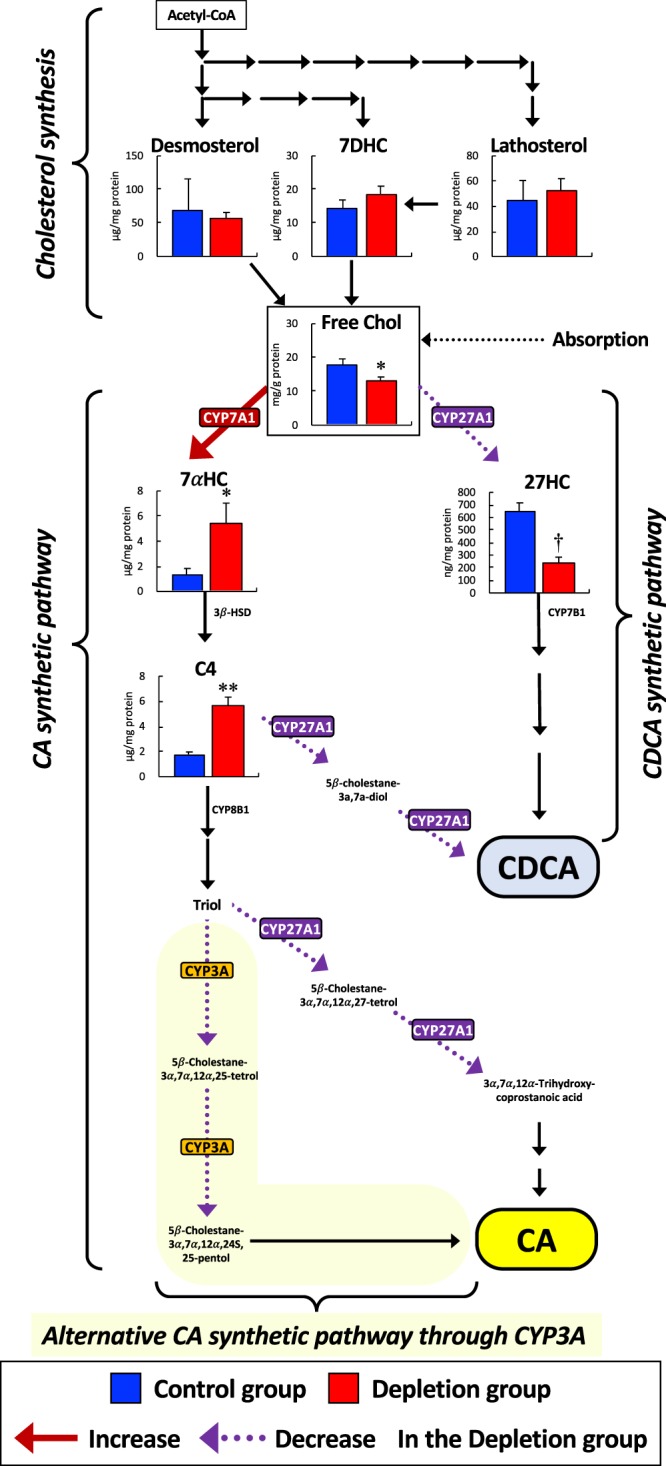
Free-cholesterol and oxysterol concentrations in the liver tissue. Bile acids are synthesized from cholesterol in the liver via two main pathways. In one pathway, cholesterol is initially converted to 7αHC by microsomal CYP7A1, which is the rate-limiting enzyme in BA synthesis. After several steps, the intermediate is then metabolized by mitochondrial CYP27A1 to finally yield CA. In another pathway, cholesterol is initially converted to 27HC by CYP27A1, and ultimately metabolized to CDCA. CDCA is also yielded by CYP27A1 from intermediates (e.g., 7αHC and C4) in the CA synthetic pathway. 7αHC, C4, and 27HC are the key intermediates through CYP7A1 and CYP27A1 in BA synthetic pathway. Lathosterol, 7-DHC, and Desmosterol are the intermediates in cholesterol synthesis. Data are the mean ± SE. *P < 0.05, **P < 0.01, ^†^*P* < 0.001 versus the Control group by unpaired Student *t*-test. *Abbreviations*: 3*β-HSD*; 3β-hydroxysteroid dehydrogenase, *7αHC*; 7α-hydroxycholesterol, *7DHC*; 7-dehydroxycholesterol, *27HC*; 27-hydroxycholesterol, *C4*; 7α-hydroxy-4-cholesten-3-one, *CYP7A1*; cholesterol 7 alpha-hydroxylase, *CYP7B1*; 25-hydroxycholesterol 7-alpha-hydroxylase, *CYP8B1*; 7α-hydroxy-4-cholesten-3-one 12α-hydroxylase, *CYP27A1*; cholesterol 27-hydroxylase, *Free Chol*; free-cholesterol, *Triol;* 5β-cholestane-3α,7α,12α-triol, and see the legend of Fig. 2 for other abbreviations.

Serum levels of α-hydroxy-4-cholesten-3-one (C4), a specific marker of microsomal cholesterol 7α-hydroxylase (CYP7A1) activity^35^, and sitosterol, a marker of intestinal cholesterol absorption^36^, were significantly increased and decreased, respectively, in the Depletion group, compared to the Control group (Table 1). In the liver, the free-cholesterol and 27-hydroxycholesterol (27HC) levels were significantly lower in the Depletion group than in the Control group (Fig. 4). However, the 7α-hydroxycholesterol (7αHC) and C4 levels were more than two-fold higher in the Depletion group than in the Control group (Fig. 4). The hepatic concentrations of lathosterol, 7-dehydroxycholesterol (7DHC), and desmosterol, which are intermediates in cholesterol synthesis pathway, were not significantly different between the two groups (Fig. 4).

### Protein expression and taurine-modified mt-tRNA levels, and mitochondrial activity

**Figure 5:**
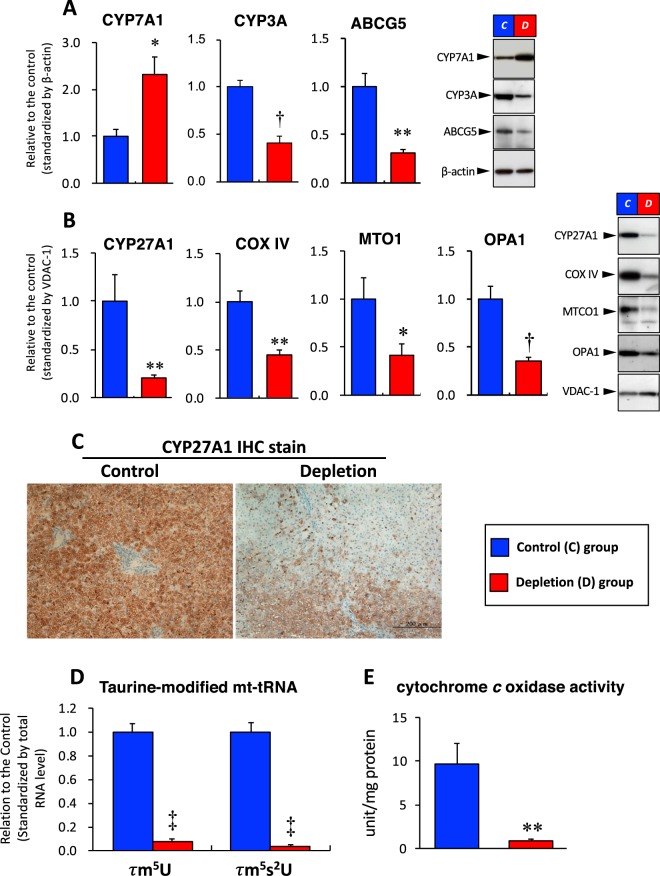
Bile acid synthesis-related protein expressions, taurine modified mt-tRNA levels and mitochondrial activity in the liver tissue. (**A,B**) The BA synthesis proteins expressed in the cytosolic (**A**) and mitochondrial (**B**) fractions analyzed by Western blotting. The protein expressions in the cytosolic and mitochondrial fractions were standardized by β-actin and VDAC-1 proteins, respectively. (**C**) Immunohistochemical stain of CYP27A1 in the liver tissue. (**D**) *τ*m^5^U and *τ*m^5^S^2^U levels in the liver. (**E**) Cytochrome *c* oxidase activity in the liver. The mt-tRNA level and mitochondrial activity were expressed as per the protein level. Data are presented as the mean ± SE. *P < 0.05, **P < 0.01, ^†^*P* < 0.001, ^†^*P* < 0.001versus the Control group by unpaired Student *t*-test. *Abbreviations*: *COX-IV*; cytochrome *c* oxidase subunit 4, *mt-tRNA*; mitochondrial-tRNA *τm*^5^*U*; 5-taurinemethyluridine, *τm*^5^*S*^2^*U*; 5-taurine*m*ethyl 2-thiouridine, and see the legends of Figs. 2 and 4 for other abbreviations.

In the liver of the Depletion group, cytoplasmic CYP7A1 protein level was significantly increased, while CYP3A (cytochrome P450, family 3, subfamily A) and ABCG5 (ATP–binding cassette subfamily G Member 5) protein levels were significantly decreased, compared to their levels in the Control group (Fig. 5A). The expressions of cholesterol 27-hydroxylase (CYP27A1), mitochondrial cytochrome *c* oxidase subunit 4 (COX-IV), mitochondrial tRNA translation optimization 1 (MTO1), and optic atrophy type 1 (OPA1) in the mitochondrial proteins were significantly decreased in the Depletion group, compared to their expression the Control group (Fig. 5B). Immunohistochemical staining revealed that CYP27A1 protein was uniformly expressed throughout the hepatic lobule in the Control group, whereas the expression of CYP27A1 was remarkably reduced in extensive areas of the pericentral regions in the Depletion group (Fig. 5C**)**.

In the Depletion group, the *τ*m^5^U and *τ*m^5^s^2^U levels in the hepatic tissue were significantly decreased to <10%, compared to their levels in the Control group (Fig. 5D). In addition, cytochrome *c* oxidase activity in the liver was significantly decreased in the Depletion group, compared to the Control group (Fig. 5E).

## Discussion

**Figure 6:**
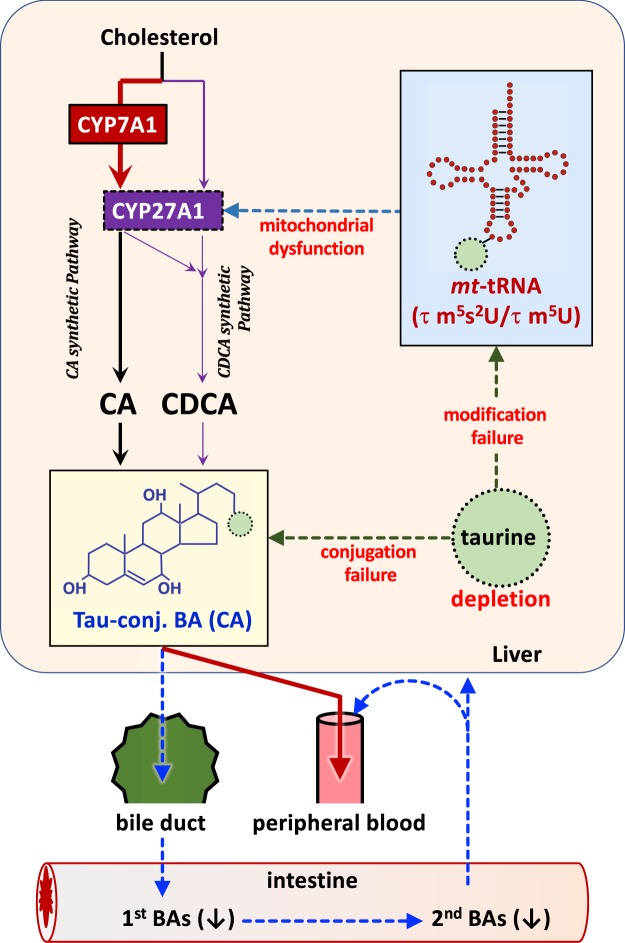
Defective BA metabolism in taurine depletion by impairments in mt-tRNA modification and in BA conjugation. Taurine depletion decreases the taurine modified mt-tRNAs, and in turn, CYP27A1 protein located on the mitochondrial inner membrane is decreased. Bile acid syntheses, particularly in CDCA synthesis, are decreased according to the accumulated substrates and decreased metabolites of CYP27A1. Bile acid excretion into bile is decreased, and instead, the excretion into the peripheral circulation is increased.

In this study, the profiling of BAs and oxysterols, the expression of enzymes involved in BA synthesis, and the levels of taurine-modified mt-tRNA were evaluated in the bile, serum, and liver of taurine-depleted cats. The taurine-deficient diet completely depleted taurine in the serum and liver of cats and, consequently, significantly decreased the levels of biliary taurine-conjugated BAs and hepatic taurine-modified mt-tRNA (Fig. 6). In BA metabolism, the expressions of the mitochondrial enzyme CYP27A1 and its metabolite 27HC in the liver were significantly decreased. The TBA level and the proportion of CDCA were consequently significantly decreased in the bile of the taurine-depleted cats (Fig. 6).

During taurine-deficient diet feeding, the serum taurine concentration was significantly decreased as early as the second week and undetectable by the seventh week. After taurine-deficient diet feeding for 30 weeks, taurine in the liver was also completely depleted, and the level of taurine-conjugated BAs in the bile was decreased to less than one-half and >60% of BAs were instead the unconjugated form, compared to these levels in the taurine-supplemented diet fed cats. This evidence agrees with a previous study using the taurine depleted cats that reported the taurine-conjugated BAs in the bile were declined to only 40% compared to 98% in control cats, and unconjugated CA was increased to 54% compared to 1% in the control, but the concentration of glycine-conjugated BAs was small and not significant1y affected by taurine depletion^7^. The conjugation of taurine and glycine occurs in the final step of BA synthesis by two key enzymes—bile acid CoA synthase and bile acid CoA-amino acid *N*-acetyltransferase (BAAT)—in the peroxisome and the cytoplasm^37^. It might be associated with the affinity of BAAT is greater for taurine rather than for glycine^38^, but the certain reason is unclear.

In contrast to the alterations in bile, the serum TBA level was significantly increased >200-fold in the present taurine depleted cats compared to that in the Control level, and was primarily in the unconjugated form (Fig. 3A,B). In cats, healthy level of the serum TBA level was reported to be approximately 0.5 µM^39^, and the higher level than 20 µM was highly suggestive of hepatobiliary disease^40^. In the present study, mild infiltration of inflammatory cells was observed around the periportal region in the taurine-depleted liver, but the significant increase of BAs in the serum should be because of the excretion failure of BAs to bile because unconjugated BAs are not excreted into the bile by the bile salt export pump (BSEP)^41^. The marked elevation in the ratios of the primary BAs to the secondary BAs in the bile and peripheral blood were observed in the Depleted group compared to those in the Control group (Figs. 2E & 3E). The secondary BAs are transformed from the primary BAs in the intestine tract following the deconjugation of amino acid-conjugated BAs by intestinal bacteria, and then, are absorbed in the distal intestine by passive transport^42^. Thus, the significant decreases of the secondary BAs might be resulted from the decrease of biliary BA extraction in the taurine-depleted cats. In addition, the amino acid-conjugated BAs are reabsorbed in the distal ileum by apical sodium-dependent bile acid transporter (ASBT)^42^. Taurine conjugated BAs were kept to 40% in the bile and 5.5% in the peripheral blood in spite of the taurine depletion in the liver might be due to the enterohepatic circulation via the ASBT.

The elevation of unconjugated BAs in the serum has been observed in individuals with a genetic defect in BAAT^43^. In patients with BAAT deficiency, malabsorption of lipids and lipid-soluble vitamins is expected because amino acid-conjugated BAs have a role in facilitating lipid absorption in the intestinal tract^44^. The present study showed significant decreases in the serum level of the plant sterol sitosterol, which is a marker of cholesterol absorption^36^, in the taurine-depleted cats. Also, the protein level of ABCG5, an ABC transporter that forms a heterodimer with ABCG8 and excretes selective sterols from the liver and intestine^45^, were significantly reduced in the taurine-depleted liver, implying that the significantly decreased level of sitosterol in the serum was not due to the enhanced biliary excretion from the liver. Therefore, this study’s findings support the idea that the malabsorption of lipids and lipid-soluble nutrients can occur by congenital or acquired defects of amino acid-conjugation with BA.

The significant decreases in the serum levels of total-cholesterol and HDL-cholesterol in taurine-depleted cats was the interesting finding in the present study. Rabin *et al*. demonstrated that the molecular percentage of biliary cholesterol was higher in the taurine depleted cats than in the taurine fed control cats, but the value was minimal, representing 0.82% and 0.33%, respectively^31^. Because the biliary TBA concentration in the Depletion group was decreased to less than one-half that in the Control group, the absolute content of cholesterol in the bile was suggested to be no difference between the two groups. So, the reason for the significant decreases in the serum levels of total-cholesterol and HDL-cholesterol in taurine-depleted cats may be associated with the malabsorption of cholesterol. Taurine depletion may directly affect other tissues, but the specific reasons for decreases in serum cholesterols—particularly in HDL-cholesterol—remain to be clarified.

In the primary BA proportions in bile, opposite alterations were characteristic in the taurine-depleted cats—namely, the CA to CDCA ratio was significantly decreased. In the Western blotting and oxysterol analyses, the expression of CYP27A1 and the level of its metabolite 27HC in the liver were significantly decreased in the taurine-depleted cats. Therefore, the significant decrease in CDCA may have been because of the low expression of CYP27A1, which is located on the mitochondrial inner membrane^46^. This evidence suggested that mitochondrial abnormalities may be caused by taurine depletion.

The present study showed that *τ*m^5^U and *τ*m^5^s^2^U levels were significantly decreased in the taurine-depleted liver. This finding is the first report of defective taurine-modified mt-tRNA being caused by acquired taurine depletion, whereas defective taurine-modified mt-tRNA has previously been reported in inherited mitochondrial diseases such as MELAS, and MERRF^33^. In mitochondrial diseases, mt-DNA-encoded proteins are suppressed; as a consequence, mitochondrial oxidative stress and permeabilization of the mitochondrial inner membrane are triggered^47^. In the taurine-depleted liver in the current study, CYP27A1 and COX-IV proteins located on the mitochondrial inner membrane^46^ were significantly decreased, whereas voltage-dependent anion-selective channel protein 1 (VDAC-1) protein located on the outer membrane were maintained. Therefore, a significant decrease in CYP27A1 protein expression may be because of the disruption of the inner membrane by defective taurine-modified mt-tRNA.

However, CYP7A1 expression was significantly increased in the taurine-depleted liver. The expression of CYP7A1 is usually downregulated by BAs through the farnesoid X receptor (FXR) NR1H4 by negative feedback^48,49^. Vaquero *et al*. reported that the change of BA profile affects the control of BA metabolism through FXR activation; particularly, CDCA significantly increased the target genes of FXR in the cultured human hepatocytes^50^, because CDCA has the most potent endogenous FXR-ligand activity among the BAs^51^. In the taurine-depleted liver, the FXR-induced downregulation of CYP7A1 expression was likely attenuated owing to significantly decreased CDCA production. In addition, the intermediates 7αHC and C4 in the CA synthetic pathway were also significantly increased in the taurine-depleted liver (Fig. 4).

Downstream of C4, CYP27A1 is also involved in the 27-hydroxylation of 5β-cholestane-3α,7α,-diol and 5β-cholestane-3α,7α,12α-triol (Triol) for their metabolism to CDCA and CA, respectively. Therefore, the accumulation of the intermediates in the CA synthetic pathway should result from a significant decrease in CYP27A1 expression, and consequently decreased CDCA and CA production in the taurine-depleted liver.

In CYP27A1 disturbance, there is the possibility that another CDCA synthetic pathway may be activated by CYP3A (Fig. 4). A previous study^52^, which used *Cyp27*^*−/−*^ mice, reported that CYP3A upregulates the hydroxylation of the C25 position of Triol to yield 5β-cholestane-3α,7α,12α,25-tetrol that is finally metabolized to CA. In contrast to the *Cyp27*^−/−^ mice, CYP3A upregulation is not induced in patients with cerebrotendinous xanthomatosis (CTX), which results from an inherited mutation of the *CYP27* gene with an excessive accumulation of intermediates in BA synthesis in various tissues and manifested by tendon xanthomatosis, premature atherosclerosis, cholesterol gallstones, osteoporosis, and progressive neurologic dysfunction^52^. Similar to CTX patients, the alternative pathway by CYP3A may not have been upregulated in the present taurine-depleted liver because of the significantly decreased CYP3A expression. On account that CYP3A expression is upregulated by the pregnane-activated receptor (PXR) NR1I2 and that LCA has the most potent ligand activity for PXR among the usual BAs^53^, the decreased CYP3A expression may be because of significantly decreased levels of LCA in the bile and the peripheral blood. The present findings suggested that the events in CTX patients may occur, at least in liver tissue, by acquired taurine depletion. Further study is needed to determine whether these CTX-like events could be caused in the whole body by acquired taurine depletion.

A limitation in the present study was that more target gene expressions of nuclear receptors could not be evaluated. The reason was that the nucleotide sequences of target genes in cats remain in the predicted stage. Therefore, adequate primers could not be designed for PCR analysis. Bile acids and their precursor oxysterols have ligand actions for the FXR, PXR, and liver X receptor that regulate the gene transcriptions related to the lipid metabolisms in various cells. Similar to the observations in other taurine depletion models^17–20,54^, the more than 200-fold elevation in the BA level circulating in the peripheral blood may have induced dysfunctions and morphological defects in the whole body in the taurine-depleted cats in the present study.

In humans, the conjugation ratio of taurine and glycine to BA is 1: 3^55^, while most BAs in cats, as well as in the rodents, are conjugated with taurine^30^. Thus, taurine depletion in humans might not induce severe effects on the BA conjugation and peripheral blood concentration, however, the unconjugated BAs would be increased also in humans when the taurine pool is decreased because the taurine conjugated BAs are not replaced by the glycine conjugated BA^29^. Besides, there is a high possibility that the modification of taurine to mt-tRNA would be influenced even in humans in a decrease of the taurine pool. Therefore, the present results suggest that insufficient intake of taurine that is a conditionally essential amino acid for humans would induce the impaired BA metabolism in the liver, and the consequent disorders would be led in the whole body.

In conclusion, the present study’s findings in a taurine-deficient cat model showed that taurine-deficient diet-induced acquired depletion impaired the metabolism and circulation of BAs because of deficits in taurine modification with mt-tRNA and taurine conjugation with BAs in the liver. In particular, the inhibition of BA synthesis due to defective mitochondrial CYP27A1 expression and the excretion failure of BAs into bile, along with the backflow into peripheral blood due to the decreased taurine-conjugated BA, appears to be critical events occurring with taurine depletion. The present results in cats suggested that insufficient taurine supplementation may trigger various diseases through abnormal BA metabolism, even in humans.

## Material and Methods

### Materials

3-Aminopyridyl-*N*-hydroxysuccinimidyl carbamate (APDS), acetonitrile, amino acids mixture standard solutions (Type B, AN-2), ammonium acetate, APDSTAG Wako Amino Acids Internal Standard (IS) mixture solution, APDSTAG Wako Eluent, dicalcium phosphate, ethanol, formic acid, methanol, potassium phosphate, sodium borate buffer, sucrose, and taurine were purchased from FUJIFILM Wako Pure Chemical Corporation (Osaka, Japan).

Cholic acid, glycocholic acid (GCA), taurocholic acid (TCA), CDCA, glycochenodeoxycholic acid (GCDCA), taurochenodeoxycholic acid (TCDCA), DCA, glycodeoxycholic acid (GDCA), taurodeoxycholic acid (TDCA), LCA, glycolithocholic acid (GLCA), taurolithocholic acid (TLCA), UDCA, glycoursodeoxycholic acid (GUDCA), 4β-hydroxycholesterol (4βHC), 7αHC, 24S-hydroxycholesterol (24SHC), 25-hydroxycholesterol (25HC), C4, lathosterol, 7DHC, desmosterol, and coprostanol were obtained from Steraloids, Inc. (Newport, RI, USA). [^2^H_4_]Cholic acid, [^2^H_4_]DCA, and [^2^H_4_]LCA were purchased from C/D/N Isotopes, Inc. (Pointe-Claire, Quebec, Canada). [^2^H_4_]Taurocholic acid was obtained from Merck, KGaA (Darmstadt, Germany). Research Laboratory of Nippon Kayaku Co. (Tokyo, Japan) supplied [^2^H_4_]CDCA. Tauroursodeoxycholic acid (TUDCA), [^2^H_4_]UDCA and [^2^H_4_]TUDCA were supplied by Tokyo Tanabe Company (Tokyo, Japan). [^2^H_7_]4β-Hydroxycholesterol, [^2^H_6_]24-hydroxycholesterol, [^2^H_3_]25HC, [^2^H_6_]desmosterol, and [^2^H_7_]sitosterol were obtained from Avanti Polar Lipids (Alabaster, AL, USA). 27-Hydroxycholesterol, [^2^H_7_]27HC, and [^2^H_7_]7αHC were prepared, as previously described^56^. Sitosterol was kindly supplied by Dr. S. Shefer (University of Medicine and Dentistry of New Jersey–New Jersey Medical School, Newark, NJ, USA).

Rabbit polyclonal anti-CYP7A1, anti-MTO1, and anti-OPA1 antibodies; rabbit monoclonal anti-CYP27A1 and anti-ABCG5 antibodies; and mouse monoclonal anti-COX-IV were purchased from Abcam (Cambridge, UK). Rabbit polyclonal anti-VDAC-1 antibody and mouse monoclonal anti-β-actin antibody were obtained from Merck, KGaA. Goat polyclonal anti-CYP3A antibody was purchased from Santa Cruz Biotechnology, Inc. (Dallas, TX).

### Cat model of taurine deficiency

Ten domestic cats were bred in the animal center of the in Ibaraki Prefectural University of Health Sciences (Ibaraki, Japan). They were randomly divided into the Control group (*N* = 4 [male:female ratio, 2:2]) and the Depletion group (*N* = 6 [male:female ratio, 3:3]). The total number of animals was referenced to the previous reported cat model^21,22^. In particular, the number of cats in the Depletion group was set a higher than in the depleted group than in the control group, because severe damages to various tissues including retina and heart were reported to be caused by taurine depletion^21,22^. Body weight and age (in days) were not significantly different between the Control and Depletion groups before the experiment (BW [mean ± standard error]: 2.9 ± 0.8 kg vs. 3.1 ± 0.4 kg; age: 479 ± 100 days vs. 414 ± 52 days). For 30 weeks, the Control and Depletion groups were fed a taurine-supplemented diet and a taurine-deficient diet, respectively. The duration of diet feeding was referenced to the previous reported cat model^21,22^, and the experimental diets were prepared based on previously described methods^57,58^ (Supplemental Table 1). The lack of taurine content in the deficient diet was confirmed after preparation. One hundred grams (dried weight) were fed daily to each cat were kept as one per cage. The cats ate all of the fed diet daily, and could access freely to water. Body weight and serum taurine levels were monitored once weekly.

After the 30-week feeding period, cats were euthanatized by an overdose injection of pentobarbital. Blood was collected from the inferior vena cava and the portal vein. Bile was collected from the gall bladder. The liver, heart, and spleen were collected and weighed. The liver tissue was maintained at −80 °C until analysis. The liver and heart were fixed with a 4% paraformaldehyde solution and embedded on paraffin for histological staining. Serum from the inferior vena cava (*i.e.*, peripheral blood) was used for the analyses of biochemical parameters, BAs, oxysterols, and amino acids, whereas serum from the portal vein was used for BA analysis. The animal experiment was performed in accordance with institutional, science community, and national guidelines for animal experimentation, and conducted with the approval of the Animal Care Committee in Ibaraki Prefectural University of Health Sciences (Ibaraki, Japan) (Permit No. 2015-21, 2016-7).

### Serum biochemical parameters

Serum ALT and AST levels were analyzed, as previously described^59,60^. Serum concentrations of glucose, FFA, total-cholesterol, HDL-cholesterol, triglyceride, total protein, phospholipids, and ALP were measured using commercially available assay kits by FUJIFILM Wako Pure Chemical Corporation (Glucose CII-test Wako, Cholesterol E-test Wako, HDL-cholesterol E-test Wako, NEFA C-test Wako, Triglyceride E-test Wako, Phospholipid C-test Wako, and LabAssay ALP).

### Taurine measurement

Taurine and glycine in the serum and liver were quantified by a derivatization method with APDS by using a high-performance liquid chromatography-electrospray ionization tandem mass spectrometry (HPLC-ESI-MS/MS) system consisting of a TSQ Vantage triple-stage quadrupole mass spectrometer (Thermo Fisher Scientific, Waltham, MA, USA) equipped with an HESI-II probe and the Prominence ultra-fast liquid chromatography system (Shimadzu, Kyoto, Japan)^61,62^.

The liver tissue was homogenized with 10-fold volume of phosphate-buffered saline (PBS), and centrifuged at 3,500 × *g* for 10 min at 4 °C to collect the supernatant. Fifty microliters of serum and 5 µL of the tissue supernatant were mixed with 50 µL of APDSTAG IS mixture (FUJIFILM Wako Pure Chemical Corporation) and 100 µL of acetonitrile. It was then centrifuged at 20,000 × *g* for 10 min. Twenty microliters of the supernatant were mixed with 20 µL of APDS-acetonitrile solution (20 mg/mL) and 60 µL of 0.2 M sodium borate buffer at pH 8.8. It was then incubated at 55 °C for 10 min. The reaction mixture was thereafter added to 100 µL of 0.1% formic acid-water solution, and 5 µL was injected into the HPLC-ESI-MS/MS system. The APDS-derivatized amino acids were separated by using a 100 mm × 2.0 mm i.d. Wakosil-II 3C8-100HG column (particle size 3 μm; FUJIFILM Wako Pure Chemical Corporation) for the analytical column and the 10 mm × 1.5 mm i.d. Wakosil-II 3C8-100HG column for the guard column (particle size 3 μm; FUJIFILM Wako Pure Chemical Corporation) at a gradient flow of 0.3 mL/min at 40 °C. The general HPLC and MS/MS conditions were conducted by using a previously described method^61^.

### Bile acids analysis

Bile acids in serum and bile were analyzed by using an HPLC-ESI-MS/MS system, based on the method described by Murakami *et al*.^63^. The IS mixture consisted of [^2^H_4_]CA (41.6 ng), [^2^H_4_]CDCA (57.5 ng), [^2^H_4_]DCA (32.8 ng), [^2^H_4_]LCA (22.4 ng), [^2^H_4_]UDCA (34.4 ng), [^2^H_4_]TUDCA (34.4 ng), [^2^H_3_]TCA (100 ng). Ninety percent ethanol-water (20 µL) was added to 20 µL of serum and to 20 µL of bile that had previously been diluted 1,000-fold with distilled water. Samples were diluted with 2 mL of 0.5 M potassium phosphate buffer (pH 7.4) and passed through Bond Elut C18 cartridges (200 mg; Agilent Technologies, Santa Clara, CA, USA)^64^. After washing the cartridge with 1.6 mL of water, BAs were eluted in 3 mL of 90% ethanol. The eluate was evaporated to dryness at 100 °C under a nitrogen stream and redissolved in 20 mM ammonium acetate buffer (pH 7.5)-methanol (1:1, *v/v*). After centrifugation at 12,000×*g* for 1 min, an aliquot of the supernatant was injected into the HPLC-ESI-MS/MS system for analysis. Chromatographic separation was conducted by using a Hypersil GOLD column (150 mm × 2.1 mm, 3-µm particles; Thermo Fisher Scientific) with a flow gradient of 200 µL/min at 40 °C. The general HPLC and MS/MS conditions were previously described^63^.

Bile acid concentrations are presented as the sum of all types of BAs conjugated with and without taurine or glycine. Amino acid conjugation of BAs is presented as unconjugated, taurine-conjugated, and glycine-conjugated in the sum of all types of BAs. The composition of BAs is presented as the proportion of CA, CDCA, DCA, LCA, and UDCA in the sum of the unconjugated and conjugated forms.

### Oxysterol analysis

Oxysterols in the liver tissue and serum were simultaneously quantified by using the HPLC-ESI-MS/MS system, as described in our previous reports^65–67^. In the liver, 27HC, 7αHC, C4, 4βHC, and 24SHC (*i.e*., the intermediates in BA synthetic pathway) and lathosterol, 7DHC, and desmosterol (*i.e*., the intermediates in the cholesterol synthetic pathway) were determined (see the schematic in Fig. 4). In the serum, the following were measured: the plant sterol sitosterol, a marker of intestinal cholesterol absorption; 4βHC, a blood marker of CYP3A activity; 25HC, a metabolite of CYP3A; and C4, a blood marker of CYP7A1 activity.

In brief, 10 µL of serum and liver tissue homogenized with 10-fold volume of PBS were added to coprostanol and deuterated oxysterols as the IS. Alkaline hydrolysis was performed in 1 N ethanolic potassium hydroxide (KOH) with butylated hydroxytoluene at 37 °C for 1 hour. Sterols were extracted with *n*-hexene, and derivatized to picolinyl esters. 7α-Hydroxy-4-cholesten-3-one was determined without alkaline hydrolysis^68^. Deuterium-labeled C4 was added to 20 µL of serum, extracted with acetonitrile, and then derivatized into picolinyl ester. All samples were injected into the HPLC-ESI-MS/MS system. All samples were thawed for immediate analysis and subsequently were not used. For purification, all samples were maintained in nitrogen to avoid autoxidation during the assays. Sitosterol content in the experimental diets was also measured by same method. Free-cholesterol concentration in the homogenized liver tissue was measured using a commercially available assay kit (Free-Cholesterol E-test Wako; FUJIFILM Wako Pure Chemical Corporation).

### Western blotting analysis

Cytosolic and mitochondrial fractions were separated by using a previously described method^56^. Liver tissue was homogenized by using a loose-fitting teflon pestle in four volumes of 3 mM Tris-HCl buffer (pH 7.4) containing 0.25 mM sucrose and 0.1 mM ethylenediamine tetra-acetic acid. The homogenate was centrifuged at 700 × *g* for 10 min and the supernatant was centrifuged again at 7,000 × *g* for 20 min. The pellet was resuspended with the buffer and used as the mitochondrial fraction. The supernatant was further centrifuged at 105,000 × *g* for 90 min and the final supernatant was the cytosolic fraction. The protein samples were boiled for 10 min with a dithiothreitol-containing sample buffer (EzApply; ATTO Corporation, Tokyo, Japan), and were separated by electrophoresis on a 5–20% gradient sodium dodecyl sulfate-polyacrylamide gel (ATTO Corporation) and transferred to polyvinylidene fluoride membranes (Immobilon P; Merck KGaA). Blots applied from the cytoplasm fraction were incubated with the primary antibodies against CYP7A1 (1:1,000), CYP3A (1:200), ABCG5 (1:1,000), and β-actin (1:5,000). Blots from the mitochondrial fraction were incubated with the primary antibodies against CYP27A1 (1:1,000), COX-IV (1:1,000), MTO1 (1:500), OPA1 (1:800), and VDAC-1 (1:1,000). The blots of the cytoplasm fraction and mitochondrial fraction were further incubated with the respective secondary antibodies (Amersham; GE healthcare, Chicago, IL, USA). The bands were visualized with an enhanced chemiluminescence detection system (Amersham), and quantified by densitometric assay using Image J software (NIH, MD, USA). The cytoplasmic CYP7A1, CYP3A, and ABCG5 protein levels were expressed as the ratio of β-actin. The mitochondrial CYP27A1, COX-IV, MTO1, and OPA1 proteins were expressed as the ratio of VDAC-1.

### Histological analysis

The liver and heart tissues were stained with H&E. Immunohistochemical (IHC) staining for the CYP27A1 protein in liver tissue was conducted by using an autostain system with exclusive reagents (Discovery XT system; Ventana–Roche Diagnostics K.K., Basel, Switzerland). Deparaffinized 5-µm thick specimens of hepatic tissue were incubated with the primary antibody for anti-CYP27A1 (1:100) for 1 hour at room temperature, and then incubated with a biotin-conjugated universal secondary antibody (Ventana) for 32 min at 37 °C. It was developed using the DAB map kit (Ventana). The nucleus and cytoplasm were stained by using Hematoxylin Counterstain (Ventana) and a bluing reagent (Ventana). Specific immunoreaction of the primary antibody was confirmed by incubation without the primary antibody.

### Taurine-modified mt-tRNA analysis

Taurine modification of mt-tRNA was examined by using mass spectrometry, as previously described^34^. In brief, total RNA was isolated from cat liver tissues by using TRIzol reagent, based on the manufacturer’s instruction. Five milligrams of total RNA were digested with 2.5 U of Nuclease P1 (FUJIFILM Wako Pure Chemical Corporation) and 0.2 U of bacterial ALP (TAKARA Bio. Inc., Shiga, Japan) in 5 mM ammonium acetate and 20 mM 4-(2-hydroxyethyl)-1-piperazineethanesulfonic acid–potassium hydroxide (pH 7.0) for 3 hours at 37 °C. A 2-mL sample of the digested total RNA sample was subjected to ultrahigh-pressure liquid chromatography coupled with triple quadrupole mass spectrometry (LCMS8050; Shimadzu). 5-Taurinemethyluridine and *τ*m^5^s^2^U were detected by using the multiple reaction monitoring method in the negative ion mode. The abundance of *τ*m^5^U and *τ*m^5^s^2^U was normalized to the abundance of unmodified adenosine.

### Mitochondrial activity in the liver

Mitochondrial activity in the liver was evaluated, based on cytochrome *c* oxidase activity, by using a commercially available kit (BioChain, Newark, CA). Mitochondria were isolated from liver tissue using a mitochondrial isolation kit (MitoCheck; Cayman Chemical, Ann Arbor, MI, USA), based on the manufacturer’s instruction. The protein content of the isolated mitochondrial sample was measured using a BCA protein assay kit (Pierce; Thermo Fisher Scientific). The mitochondrial activity is presented per protein.

### Statistical analysis

Statistical significance was determined by using Student’s *t*-test. Each value is expressed as the mean ± the standard error (SE). Differences were statistically significant when the calculated *p*-value was less than 0.05. Statistical analyses were conducted using JMP software (version 14.3, SAS Institute; Cary, NC, USA).

## Supplementary information

Supplemental Table & Figs.
