# Supplementary material for: Impaired bile acid metabolism with defectives of mitochondrial-tRNA taurine modification and bile acid taurine conjugation in the taurine depleted cats
Source: Sci Rep. 2020 Mar 18;10:4915. doi: 10.1038/s41598-020-61821-6 (PMC7080809; doi:10.1038/s41598-020-61821-6)
Supplement: Supplementary file 1 — Supplemental Table & Figs. [file 41598_2020_61821_MOESM1_ESM.pdf]

Supplemental Materials

Impaired bile acid metabolism with defectives of mitochondrial-tRNA taurine modification and bile acid taurine conjugation in the taurine depleted cats

Teruo Miyazaki[1,*], Sei-Ich Sasaki[2,3], Atsushi Toyoda[4], Fan-Yan Wei[5], Mutsumi Shirai[1], Yukio Morishita[6], Tadashi Ikegami[7], Kazuhito Tomizawa[5], Akira Honda[1,7]

1 Joint Research Center, Tokyo Medical University Ibaraki Medical Center, Ibaraki, 300-0395, Japan

2 Ibaraki Prefectural University of Health Sciences, Ibaraki, 300-0394, Japan

3 Toyo Public Health College, Tokyo, 151-0071, Japan

4 College of Agriculture, Ibaraki University, Ibaraki, 300-0393, Japan

5 Department of Molecular Physiology, Faculty of Life Sciences, Kumamoto University, Kumamoto, 860-8556, Japan

6 Diagnostic Pathology Division, Tokyo Medical University Ibaraki Medical Center, Ibaraki, 300-0395, Japan

7 Department of Internal Medicine, Division of Gastroenterology and Hepatology, Tokyo Medical University Ibaraki Medical Center, Ibaraki, 300-0395, Japan

*Corresponding Author:

E-mail: teruom@tokyo-med.ac.jp

Supplemental Table 1. Composition of the experimental diets

Contents	Taurine-supplemented diet	Taurine-deficient diet
Soy protein isolate	409 g	411 g
Corn starch	202 g	202 g
Sucrose	200 g	200 g
Beef tallow	100 g	100 g
AIN 93G mineral mix	50 g	50 g
Dicalcium phosphate	24 g	24 g
AIN 93 vitamin mix	10 g	10 g
Choline chloride	3 g	3 g
Taurine	1.5 g	*Absent*
total	1,000 g	1,000 g
Sitosterol content	1.75 mg/1,000g	1.75 mg/1,000g

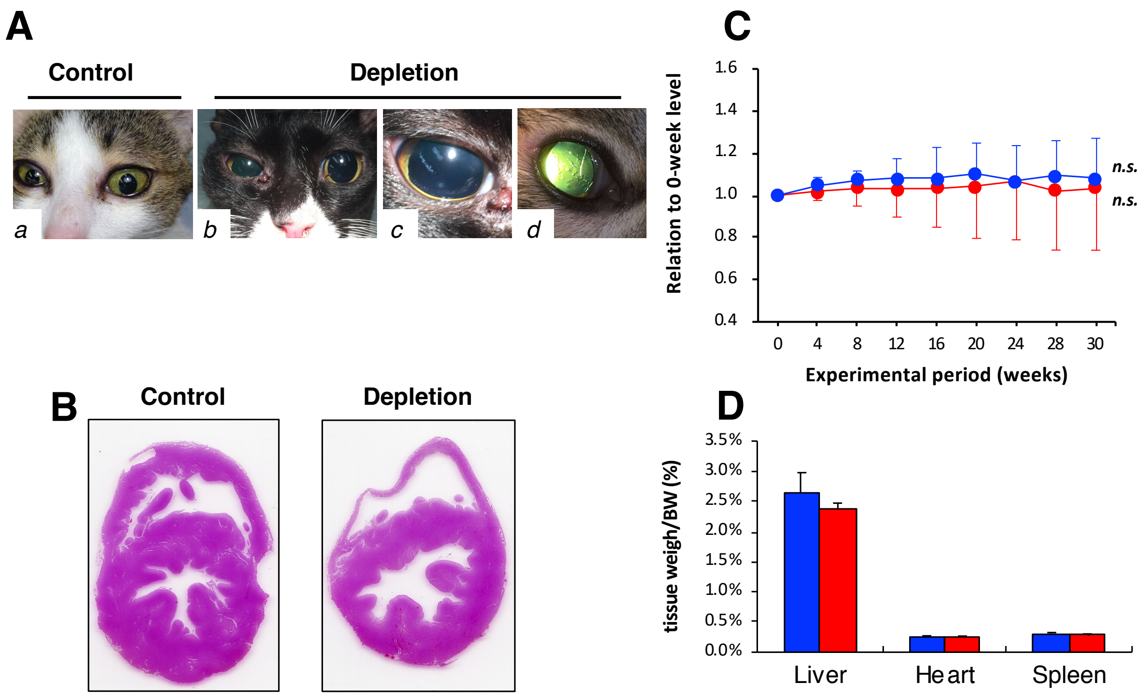

Supplemental Fig. 1. Fig.S1A: Pictures of eyes at the terminal of the taurine-deficient diet feeding period for 30 weeks. (*a*) Control cat, (*b*) Taurine-depleted cat, (*c*) Enlarged picture of right eye in picture (b), and (*d*) Enlarged picture of eye with light in another taurine-depleted cat. **Fig. S1B**: Overall of histological image in H&E stain of heart. **Fig. S1C**: Progress of BW change during the feeding period. The BW change is presented as the rate to the respective value before the feeding. **Fig. S1D:** Tissue weights of liver, spleen, and heart after the feeding period.

A. Bile acid concentrations in bile

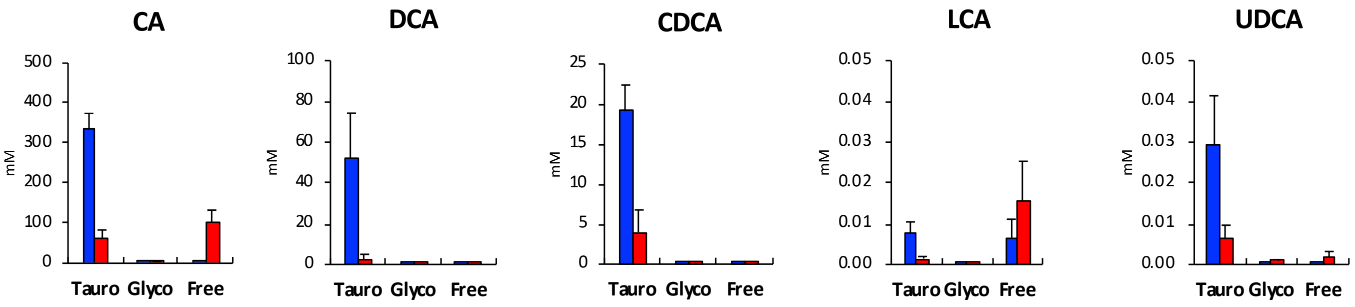

B. Composition of bile acids in bile

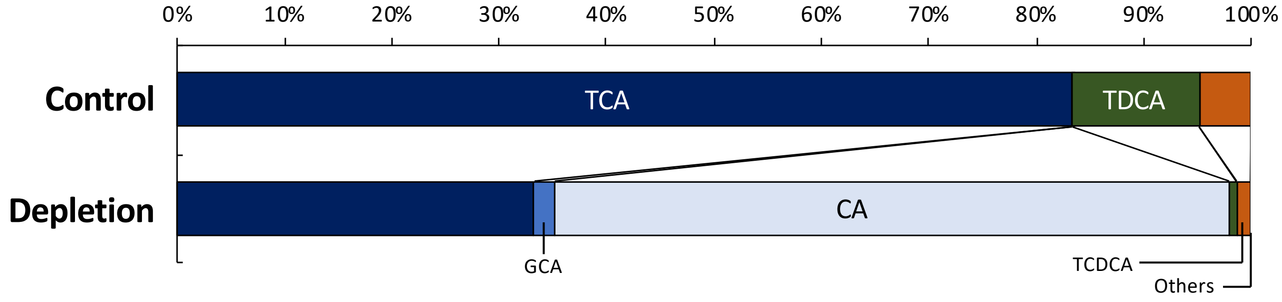

Supplemental Fig. 2. Bile acids concentrations and composition of all types and conjugation forms, and compositions of bile acid conjugation forms in bile. ***A:*** Bile acid concentrations in bile. Tauro, Glyco, and Free show taurine-conjugated, glycine-conjugated, unconjugated bile acids, respectively. Blue and red columns show the Control and Depletion groups, respectively. Data are shown as the mean ± SEM. ***B:*** Composition of bile acids in bile.

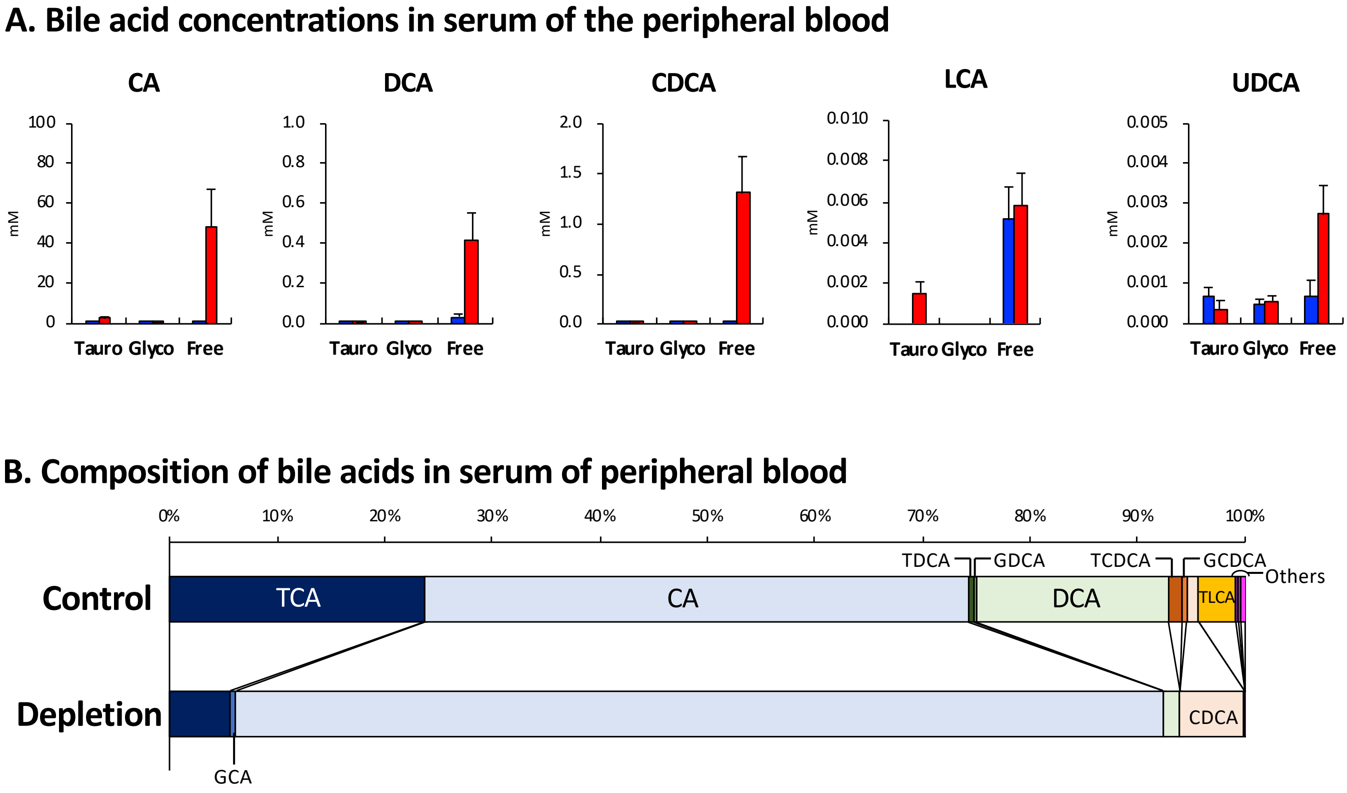

Supplemental Fig. 3. Bile acids concentrations and composition of all types and conjugation forms, and compositions of bile acid conjugation forms in serum of the peripheral blood. ***A:*** Bile acid concentrations in serum of the peripheral blood. Tauro, Glyco, and Free show taurine-conjugated, glycine-conjugated, unconjugated bile acids, respectively. Blue and red columns show the Control and Depletion groups, respectively. Data are shown as the mean ± SEM. ***B:*** Composition of bile acids in serum of the peripheral blood.

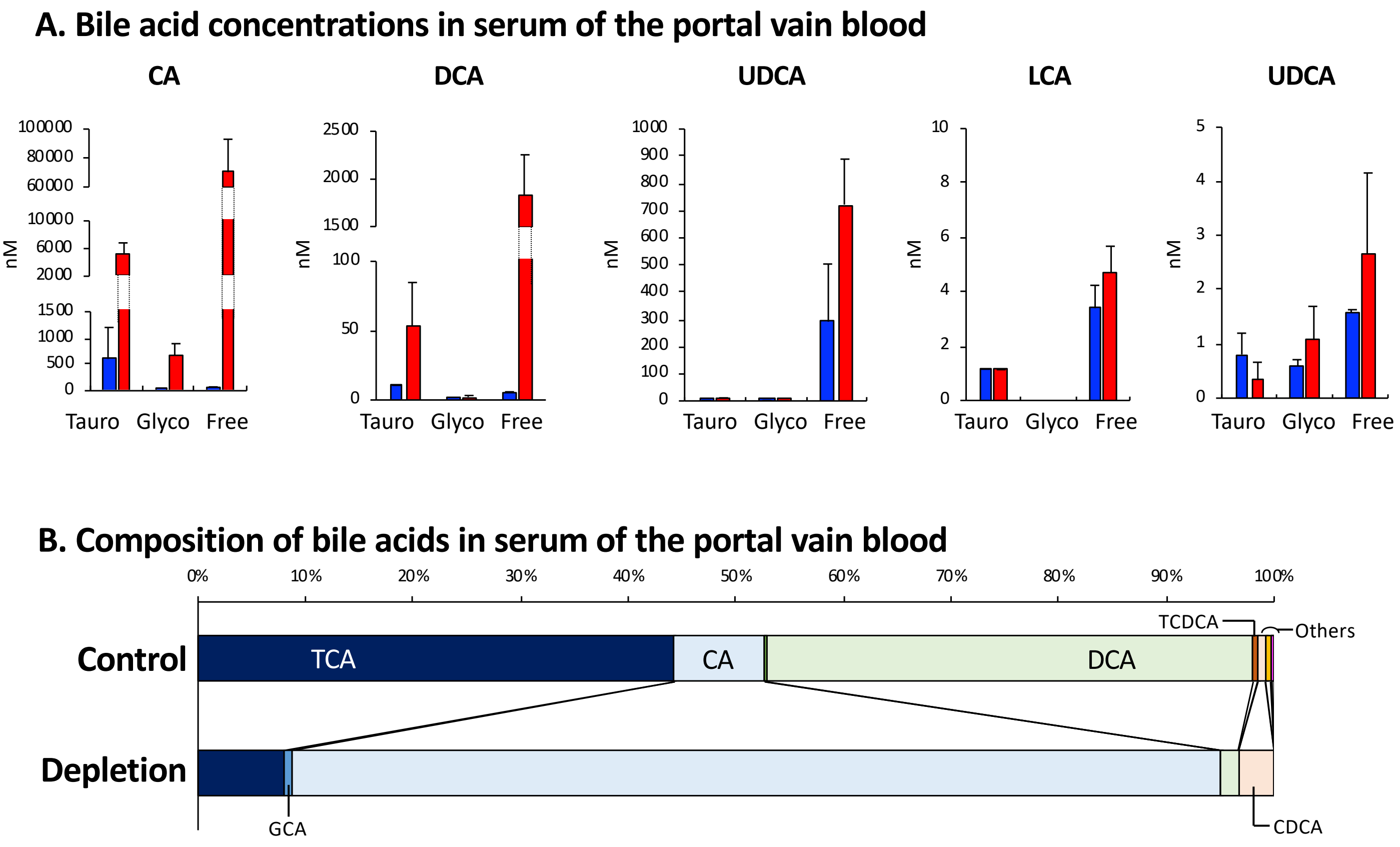

Supplemental Fig. 4. Bile acids concentrations and composition of all types and conjugation forms, and compositions of bile acid conjugation forms in serum of the portal vain blood. Blood was collected from the portal vain in the Control (*N*=2) and Depletion (*N*=4) groups. ***A:*** Bile acid concentrations in serum of the portal vain blood. Tauro, Glyco, and Free show taurine-conjugated, glycine-conjugated, unconjugated bile acids, respectively. Blue and red columns show the Control and Depletion groups, respectively. Data are shown as the mean ± SEM. ***B:*** Composition of bile acids in serum of the portal vain blood.

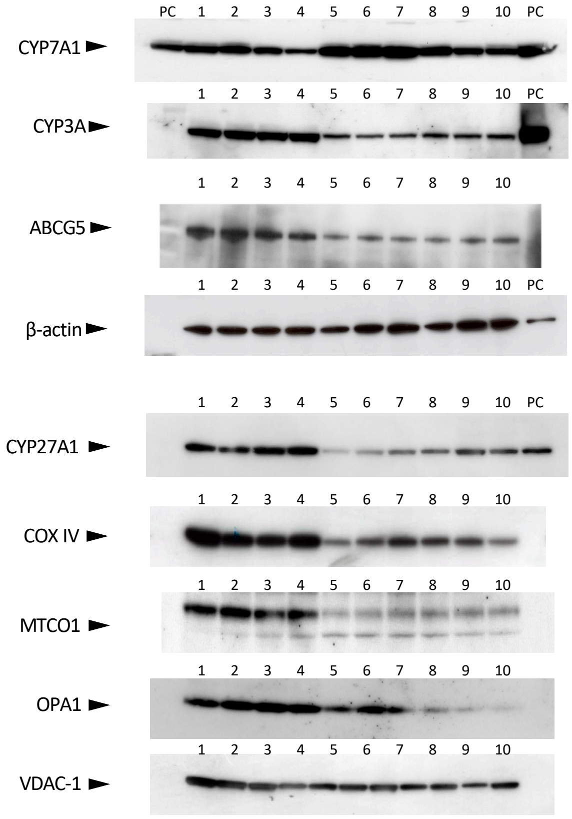

Supplemental Fig. 5. Blots of Western blotting in the cat liver. The protein expressions of CYP7A1, CYP3A, ABCG5 and β-actin were examined in the cytoplasm fraction, while the others were in the mitochondrial fractions. Blots were obtained from different membranes. Lanes 1-4; the Control group (*N*=4), Lanes 5-10; the Depletion group (*N*=6), PC; positive control obtained from rat liver.
